# Retroauricular/Transcranial Color-Coded Doppler Ultrasound Approach in Junction With Ipsilateral Neck Compression on Real-Time Hydroacoustic Variation of Venous Pulsatile Tinnitus

**DOI:** 10.3389/fnhum.2022.862420

**Published:** 2022-06-15

**Authors:** Xiuli Gao, Yue-Lin Hsieh, Xing Wang, Wuqing Wang

**Affiliations:** ^1^Department of Radiology, Eye Ear Nose & Throat Hospital, Fudan University, Shanghai, China; ^2^Department of Otology and Skull Base Surgery, Eye Ear Nose & Throat Hospital, Fudan University, Shanghai, China; ^3^NHC Key Laboratory of Hearing Medicine, Shanghai, China; ^4^School of Mechanical and Automotive Engineering, Xiamen University of Technology, Xiamen, China

**Keywords:** pulsatile tinnitus, Doppler ultrasound, transcranial Doppler (TCD) ultrasonography, sigmoid sinus wall anomalies, diverticulum, computational fluid dynamics

## Abstract

Alterations in dural venous sinus hemodynamics have recently been suggested as the major contributing factors in venous pulsatile tinnitus (PT). Nevertheless, little is known about the association between real-time alterations in hemodynamics and the subjective perception of venous PT. This study aimed to investigate the hydroacoustic correlations among diverticular vortices, mainstream sinus flow, and PT using various Doppler ultrasound techniques. Nineteen venous PT patients with protrusive diverticulum were recruited. The mainstream sinus and diverticular hemodynamics before and after ipsilateral internal jugular vein (IJV) compression were investigated using an innovative retroauricular color-coded Doppler (RCCD) method to examine the correlation between the disappearance of PT and hemodynamic alterations. To reveal the hydroacoustic characteristics of disparate segments of venous return, a computational fluid dynamics (CFD) technique combined with the transcranial color-coded Doppler method was performed. When the ipsilateral IJV was compressed, PT disappeared, as the mean velocity of mainstream sinus flow and diverticular vortex decreased by 51.2 and 50.6%, respectively. The vortex inside the diverticulum persisted in 18 of 19 subjects. The CFD simulation showed that the flow amplitude generated inside the transverse–sigmoid sinus was segmental, and the largest flow amplitude difference was 20.5 dB. The difference in flow amplitude between the mainstream sinus flow and the diverticular flow was less than 1 dB. In conclusion, the sensation of PT is closely associated with the flow of kinetic energy rather than the formation of a vortex, whereby the amplitude of PT is correlated to the magnitude of the flow velocity and pressure gradient. Additionally, the range of velocity reduction revealed by the RCCD method may serve as a presurgical individual baseline curative marker that may potentially optimize the surgical outcomes.

## Introduction

Vascular pulsatile tinnitus (PT) is an abnormal perception of pulse-synchronous vascular somatosounds (Kim et al., 2016; Lee et al., 2020). Venous PT represents the largest demographic among the vascular origins of PT (Dong et al., 2015). Venous PT is characterized by the reduction or elimination of a vascular sound when the ipsilateral internal jugular vein (IJV) is compressed (Mattox and Hudgins, 2008; Grewal et al., 2014). In addition, anatomical abnormalities related to the structural integrity of the sigmoid plate and the vascular shape are often revealed using imaging modalities (Dong et al., 2015; Cummins et al., 2021; Ding et al., 2021).

Sigmoid sinus wall anomalies (SSWAs) are the most identified findings in patients with venous PT (Dong et al., 2015). SSWAs refer to sigmoid sinus wall dehiscence and sigmoid sinus diverticulum. Without the bolster of the overlying sigmoid plate, a diverticulum forms when the sigmoid sinus vascular wall protrudes into the mastoid air cells and/or the mastoid cortex (Eisenman et al., 2018; Mu et al., 2022). Given the increase in flow velocity, sinus flow carries higher kinetic energy in the transverse–sigmoid junction region after releasing from transverse sinus stenosis (TSS; Hewes et al., 2020; Zhao et al., 2021a). A high degree of TSS can further exacerbate the *trans*-stenotic pressure gradient of the transverse sinus, which impairs intracranial pressure that may be less relevant to the production of PT (Zhao et al., 2021a,b). Additionally, studies have suggested that high wall pressure and wall shear stress induced by TSS may underpin the development of SSWAs (Hsieh and Wang, 2020; Mu et al., 2022), and the recent discovery of bone remodeling of the dehiscent sigmoid plate after excluding hemodynamic pathologies *via* endoluminal stenting surgery supports this postulation (Qiu et al., 2021a).

Despite the diverse anatomical anomalies, ipsilateral IJV compression has served as a diagnostic criterion and surgical indication for venous PT (Grewal et al., 2014; Guo and Wang, 2015; Li et al., 2021). This maneuver pinpoints a cause-and-effect relationship between the restriction of venous outflow and the diminution of PT (Wang et al., 2019; Hsieh and Wang, 2020), even though the correlation between PT symptomology and sinus hemodynamics has not been quantified hitherto. In addition to the volumetric asymmetry and high sinus flow velocity that are commonly discovered in PT subjects with SSWAs, some authors also speculated that a regional complex vortical flow structure might engender PT (Hsieh and Wang, 2020; Mu et al., 2020; Li et al., 2021). Considering that (a) the velocity profile varies among individuals with venous PT and (b) vortical flow structure emanates from the transverse–sigmoid junction with or without diverticulum (Mu et al., 2020; Ding et al., 2021; Hsieh et al., 2021a; Li et al., 2021; Qiu et al., 2021b), it is, however, unclear how the velocity and diverticular vortex affect the hydroacoustic production of PT and how fluctuations in the cross-sectional area of the transverse–sigmoid sinus lumen affect the flow acoustics.

Previous studies have implemented a Doppler ultrasound to capture *in vivo* hydroacoustic characteristics of PT by sensing the bilateral IJV outflow, in which the motion of IJV outflow displayed a high degree of resemblance to the patient’s PT (Hsieh et al., 2021b). As the Doppler sampling depth and volume are adjustable, the vascular Doppler ultrasound has the advantage over unfolding the hemodynamics and flow acoustics at different regions of interest in simultaneity. Henceforth, the transcranial Doppler examination of the transverse–sigmoid sinus system may shed light on the hydroacoustic characteristics of mainstream sinus flow and diverticular vortex, which has not been attempted on subjects with venous PT in previous literature.

This study aimed to crystallize the quantitative correlation between PT and hemodynamics by combining the application of various Doppler techniques. Furthermore, we proposed the retroauricular color-coded Doppler (RCCD) ultrasound technique that allows a short-range insonation of the mainstream sinus and diverticular flows *via* the eroded mastoid cortical bone window. This technique has not been documented in previous literature and can be implemented in junction with ipsilateral IJV compression to unravel the hydroacoustic characteristics of mainstream sinus flow and diverticular vortex. Additionally, quantification of the simultaneous reduction of PT and outflow velocity may serve as a prospective indicator for surgical intervention, which can help clinicians establish the correct scope of surgery and optimize therapeutic outcomes.

## Materials and Methods

### Patient Clinical Data and Study Design

This retrospective study recruited 19 venous PT patients with a protrusive diverticulum who underwent RCCD ultrasound examination. All participants were treated at the Otology and Skull Base Surgery Center of the Eye, Ear, Nose, and Throat Hospital at Fudan University from October 2020 to April 2022. The clinical diagnosis of venous PT included prudent cervical Doppler ultrasound and IJV compression, a water occlusion test, and radiological imaging [computed tomography (CT)/contrast-enhanced CT venogram and magnetic resonance (MR) venogram/contrast-enhanced MR venogram]. The inclusion criteria were strict radiological evidence of SSWAs and a positive response to IJV compression guided by color-coded Doppler visualization. The exclusion criteria included a non-venous origin of PT (arterial and arteriovenous causes of PT), PT secondary to intracranial/cervical neoplasms, and systemic diseases such as anemia and hyperthyroidism.

**FIGURE 1:**
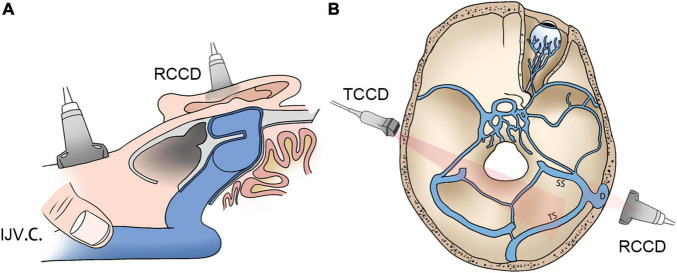
Schematic diagrams of RCCD and TCCD application. **(A)** Assessment of hydroacoustics and PT using RCCD and IJV compression. **(B)** The TCCD technique for hemodynamic assessment of contralateral transverse sinus. RCCD, retroauricular color-coded Doppler; TCCD, transcranial color-coded Doppler; and PT, pulsatile tinnitus; IJV.C., internal jugular vein compression.

Schematic diagrams of the RCCD and transcranial color-coded Doppler (TCCD) techniques and their relative anatomical structures are shown in Figure 1. The diagnosis of venous PT was ascertained by the reduction or disappearance of the PT during IJV compression. SSWAs were defined based on the criteria described by Eisenman et al. (2018): (1) dehiscence: consecutive three 0.6 mm axial CT cuts in the absence of a sigmoid plate with an osseous structure overlying the sigmoid sinus vessel wall; and (2) diverticulum: outward protrusion of an irregularly shaped sigmoid sinus vascular wall into the mastoid air cells or the mastoid cortex. In the TSS measurement, the largest and smallest cross-sectional areas of the transverse sinus lumen on each side were measured between the proximal end (the intersecting point of the confluens sinuum and the superior sagittal sinus) and the distal end (0.5 mm prior to the transverse–sigmoid junction) using MR venogram images *via* the software Mimics 19.0 (Materialise, Belgium). The normalized degree of TSS was scaled based on [Eq. (1)]:


(1) $$T\,S\,S_{n\,o\,r\,m\,a\,l\,i\,z\,e\,d}=\frac{c\,r\,o\,s\,s.a\,r\,e\,a_{m\,a\,x}-c\,r\,o\,s\,s.a\,r\,e\,a_{m\,i\,n}}{c\,r\,o\,s\,s.a\,r\,e\,a_{m\,a\,x}},$$

where cross.area_max_ and cross.area_min_ indicate the largest and smallest cross-sectional areas of the ipsilateral transverse sinus lumen, respectively.

The index of TSS (ITSS) was used based on the Carvalho criteria (Da Silveira Carvalho et al., 2017) [Eq. (2)]:


(2) I⁢T⁢S⁢S=degree⁢of⁢left⁢T⁢S⁢S⁢×⁢degree⁢of⁢right⁢T⁢S⁢S.

The degree of TSS was categorized into five distinctive scorings, where a score of 0 indicated normal, 1 indicated stenosis up to one-third (<33%), 2 indicated stenosis between one-third and two-thirds (33–66%), 3 indicated stenosis over two-thirds (>66%), and 4 indicated transverse sinus hypoplasia. Transverse sinus hypoplasia was defined as stenosis of 40% of the entire length of the transverse sinus (Durst et al., 2016).

### Retroauricular Color-Coded Doppler Technique and Modulation of Pulsatile Tinnitus/Hemodynamics

An innovative RCCD technique was proposed to examine the mainstream sinus and diverticular flow characteristics at the transverse–sigmoid junction. RCCD was performed using the LISENDO 880 ultrasound system and Arietta 60 ultrasound system (Hitachi Aloka Medical Ltd., Japan) with S121 (5–1 MHz) and L441 (12–2 MHz) transducers in a silent room. Participants were asked to lie in a supine position. The transducer was placed in the retroauricular skin tissue above the diverticulum for insonation. Prior to Doppler insonation, sonologists carefully measured the 3D shape, size, and depth of the diverticulum and its relative anatomical landmarks using CT and MR venography to localize the diverticulum.

**FIGURE 2:**
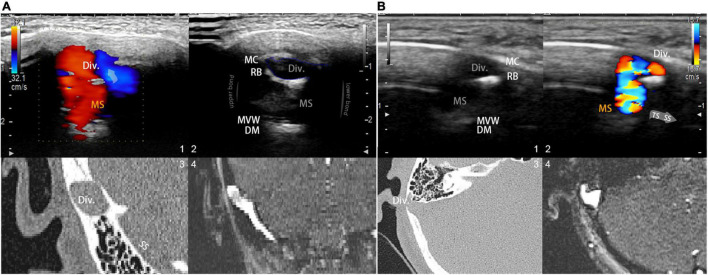
Visualization and insights of the RCCD ultrasound method. (**A**; 1) Coronal RCCD continuous wave mode view of the mainstream sinus and diverticular flows. The transparent arrow indicates mainstream sinus flow diversion into the diverticulum. Div. indicates diverticulum; MS indicates mainstream sinus flow. (2) A 2D-planar cross-sectional view of the diverticulum and the mainstream sinus anatomical structures. MC indicates mastoid cortex; RB indicates the remnant mastoid bone; MVW indicates the medial vascular wall; and DM indicates the layers of dura mater. (3) Coronal CT view of the diverticulum and sigmoid sinus corresponding to the representative 2D ultrasonographic view. (4) Coronal 2D time-of-flight magnetic resonance imaging of the diverticulum and sigmoid sinus corresponding to the representative 2D ultrasonographic view. **(B)** A 2D-planar axial view of the diverticulum and mainstream sinus anatomical structures. MC indicates the mastoid cortex; RB indicates the remnant mastoid bone; MVW indicates the medial vascular wall; and DM indicates the layers of the dura mater. (1) A 2D-planar cross-sectional view of the diverticulum and mainstream sinus anatomical structures. MC indicates the mastoid cortex; RB indicates the remnant mastoid bone; MVW indicates the medial vascular wall; and DM indicates the layers of the dura mater. (2) Axial RCCD continuous wave mode view of the mainstream sinus and diverticular flows. The gray arrow indicates the flow direction from the transverse sinus to the sigmoid sinus. Div. indicates diverticulum; MS indicates mainstream sinus flow. (3) An axial CT view of the diverticulum and sigmoid sinus corresponding to the representative 2D ultrasonographic view. (4) Axial 2D time-of-flight magnetic resonance imaging of the diverticulum and sigmoid sinus corresponding to the representative 2D ultrasonographic view.

To reveal mainstream sinus and diverticular hydroacoustics, the coronal and sagittal footage of the diverticulum can be visualized using the B-mode (flow and anatomical features are shown in Figure 2). Due to the heterogeneity of participants, a thin layer of the remnant bony structure of the mastoid may present as the second layer of high-signal intensity interpolated between the lateral diverticulum and the medial mainstream. This can be a helpful reference landmark for differentiating the diverticulum from mainstream sinus lumen. After insonating using the color mapping mode, the mainstream sinus flow and diverticular flows can be appreciated, and the range and border of the sinus flow can be cross-referenced using CT/MR venogram images. Notably, the relative position of the mainstream sinus flow and the diverticular flow depends on the anterosuperior, anteroinferior, or lateral direction of the diverticular protrusion. Once the mainstream sinus and diverticular flows were both detected in simultaneity, the location of insonation was unchanged throughout all procedures of measurement. Following the initial RCCD examination, modulation of IJV outflow was performed by the first co-author (Y-LH) to assess changes in the intrasinus hemodynamics and PT. To obtain hemodynamics when PT subsides, the Doppler velocity spectra and hemodynamic parameters were acquired after the reduction of velocity stabilized at least five cardiac cycles during the ipsilateral IJV compression. The compression time was controlled within 15 s to prevent any potential adverse effects. The sampling volume was set within the detected vessel and adjusted from 1 to 3 mm. The depth was adjusted between 3 and 4.5 cm based on the intrasubject variability. The velocity range was modified based upon the individual sinus flow velocity. The transducer frequency was 4.0∼5.22 MHz. The color-Doppler gain was then adjusted to range from 20–120 until the pulsatile flow could be fully observed, while the surrounding subcutaneous tissue remained virtually free of color. The mean flow velocity (*V*_mn_), peak velocity (*V*_max_), flow volume, resistive index, and pulsatility index at the transverse–sigmoid junction were obtained.

### Bilateral Jugular Vein Hemodynamics

The procedures used to obtain bilateral cervical hemodynamics were identical to our previously described methods (Hsieh et al., 2021b). In brief, the hemodynamics of the upper bilateral IJV were gaged at the mandibular region at the level of the skull base using a sonographic transducer L441 with a center frequency of 2–12 MHz. Bilateral hemodynamic parameters were obtained, including the *V*_mn_, *V*_max_, flow volume, resistive index, and pulsatility index.

### Transcranial Color-Coded Doppler Technique

**FIGURE 3:**
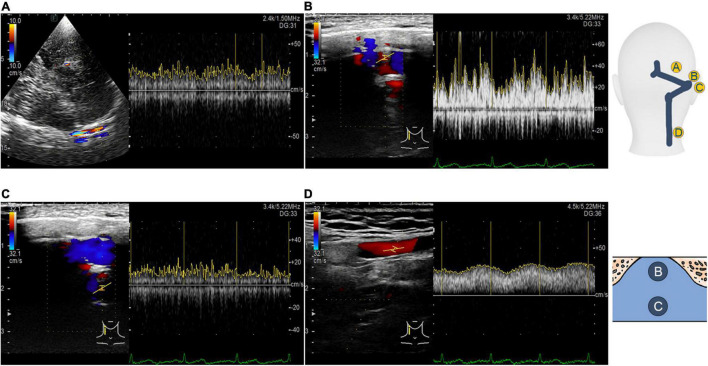
Transcranial, retroauricular, and cervical color-coded Doppler methods to assess hydroacoustics of the transverse–sigmoid sinus system. **(A)** Visualization and velocity spectrum of the proximal transverse sinus flow using the TCCD. **(B)** Visualization and velocity spectrum of the diverticular flow using the RCCD. **(C)** Visualization and velocity spectrum of the mainstream intrasinus flow using the RCCD. **(D)** Visualization and velocity spectrum of the upper internal jugular flow using the RCCD.

A LISENDO 880 ultrasound system (Hitachi Aloka Medical Ltd., Japan) with transducer S121 was used to measure the hemodynamics of the ipsilateral transverse sinus. A contralateral bone window was used to examine the ipsilateral transverse sinus. The contralateral skeletal contour became visible after increasing the depth of the B-mode. A low-flow-sensitive color program with a low-wall-filter setting was used. The depth was adjusted approximately between 10 and 15 cm based on the individual differences. The velocity range of detection was adjusted approximately from 0 to 66.84 cm/s based on individual sinus flow velocity. The sample volume was set from 1 to 3 mm based on the width of the detected vessel, as appropriate. The transducer frequency ranged approximately from 1.5 to 1.88 MHz, and the color gain was modified between 20–120 until the contour of transverse sinus flow can be fully appreciated. To locate the transverse sinus using the contralateral temporal bone window, the great cerebral vein is instantly discovered behind the pineal gland, where the rostral part of the superior sagittal sinus can be successively exposed. The straight sinus is exposed by tilting the transducer upward to align the insonation plane with the apex of the cerebellar tentorium. The torcular herophili can be found by tracing the course of the straight sinus that drains posteriorly into the occipital region. Consequently, the transverse sinus is located. Doppler examination of the ipsilateral transverse–sigmoid sinus is demonstrated in Figure 3.

### Hydroacoustic Computational Fluid Dynamics Simulation

To demonstrate and compare the ipsilateral transverse–sigmoid sinus flow and acoustic fields to the Doppler results, the computational fluid dynamics (CFD) technique was implemented in a participant with a complete proximal TCCD/RCCD sinus flow profile (case 3). Reconstruction of the participant’s 3D vascular models was based on patient-specific MR venogram images using Mimics 19.0 and 3-Matic 11.0 (Materialise, Belgium), analogous to our previously described methods (Hsieh and Wang, 2020). The contralateral transverse–sigmoid sinus and branches of the sinus were removed.

For the simulation of the flow field, a total of 456,231 elements were established using ANSA version 22.0.1 (BETA CAE Systems), in which 10 layers with a boundary layer thickness of 0.4 mm were created for the flow field. There were 63,506 elements where three layers with a boundary layer thickness of 0.1 mm were established for the acoustic simulation. The outlet pressure was set to zero. The flow velocity inlet was set based on the TCCD flow spectrum. The continuity equation and Navier–Stokes equations [Eqs (3, 4)] were solved using the transient laminar method with the software Star-CCM+ 2020 (Siemens, Germany):


(3) ∇⋅u=0

(4) $$\rho \,\frac{\partial \text{u}}{\partial t}+\rho \,\text{u}\cdot \nabla \text{u}=-\nabla \text{p}+\mu \,\nabla^{2}\text{u},$$

where **u** is the velocity vector of the incompressible Newtonian blood flow, the blood density ρ is 1,050 kg/m^3^, and the dynamic viscosity μ is 0.00345 Pa s. The results of the grid independence tests are shown in Table 1. The average velocity of the IJV outflow was selected as the criterion for generating a sufficient grid size and number. The relative error of average outflow velocity was less than 5%, which was considered acceptable for this study.

**TABLE 1:** Mesh independence analysis.

Number of volume mesh element	Element size (mm)	Average IJV outlet velocity (m/s)	Relative error (%)
325,943	0.4	2.668164e-01	0.33%
456,231	0.3	2.675777e-01	0.04%
815,281	0.2	2.676987e-01	

**FIGURE 4:**
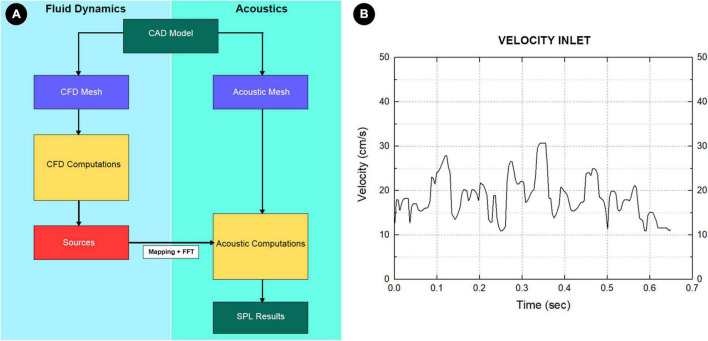
**(A)** The complete workflow of computational fluid dynamics (CFD) simulation. First, the pressure, density, and velocity fields output by the CFD solver was read. Second, the contributions according to the finite element method formulation were implemented in Actran software. Third, the contributions were projected on the acoustic mesh. Fourth, the corresponding data were stored and displayed in the Actran software. Fifth, the quantities from the time to the frequency domain were transformed using the Fourier transform algorithm. **(B)** Boundary condition at the inlet section.

Computation of the hydroacoustic source using unsteady CFD data was achieved using the software Actran 2020 (MSC, Free Field Technology, Belgium). The workflow of the CFD method, which consists of five major steps, is illustrated in Figure 4. A quadrupole sound source was set up for the transformation of the pressure fluctuation and flow velocity. For incompressible CFD computations, only the velocity field is required (Free Field Technologies, 2020). The observed frequency range was 0–1,000 Hz. The reference pressure was set at 20 μPa. Three pulsatory cycles were calculated, and the second pulsatory cycle was chosen for data presentation.

Based on the manual instruction of Actran, the implementation of variational formulations of Lighthill’s analogy derived by Oberai et al. (2000), starting with Lighthill’s equation based on Actran user’s guide (Free Field Technologies, 2020), is given as follows [Eq. (5)]:


(5) $$\frac{\partial^{2}}{\partial t^{2}}\,\left(\rho -\rho_{0}\right)-c_{0}^{2}\,\frac{\partial^{2}}{\partial x_{i}\,\partial x_{j}}\,\left(\rho -\rho_{0}\right)=\frac{\partial^{2}T_{i\,j}}{\partial x_{i}\,\partial x_{j}},$$

where *T*_*ij*_ is the Lighthill tensor [Eq. (6)]:


(6) $$T_{i\,j}=\rho \,u_{i}\,u_{j}+\delta_{i\,j}\,\left(\left(p-p_{0}\right)-c_{c}^{2}\,\left(\rho -\rho_{0}\right)\right)-\tau_{i\,j},$$

where *p* and ρ are pressure and density, respectively. The reference value in the medium at rest was ρ_0_, δ_*ij*_ is a Kronecker delta, τ_*ij*_ is the viscous stress tensor, and *c_0_* is the reference sound velocity. *T*_*ij*_ is the Lighthill tensor, and *u_i_* and *u_j_* are fluid velocity components. After a strong variational formulation of Eq. (5) and integration by parts along with spatial derivatives following Green’s theorem, the variational formulation of Lighthill’s analogy based on the fluctuation of acoustic density ρ_*a*_ = ρ−ρ_0_ was rewritten as [Eq. (7)]:


(7) $$\int_{\Omega }\left(\frac{\partial^{2}\rho_{\text{a}}}{\partial t^{2}}\,\delta \,\rho +c_{c}^{2}\,\frac{\partial \rho_{a}}{\partial x_{i}}\,\frac{\partial \delta \,\rho }{\partial x_{i}}\right)\,dx=-\int_{\Omega }\frac{\partial T_{i\,j}}{\partial x_{j}}\,\frac{\partial \delta \,\rho }{\partial x_{i}}\,dx+\int_{\partial \Omega =\Gamma }\frac{\partial \Sigma_{i\,j}}{\partial x_{j}}\,n_{i}\,\delta \,\rho \,d\Gamma \,\left(x\right),$$

where δρ is a test function, Ω represents the computational domain. The Σ_*ij*_ [Eq. (8)] is:


(8) $$\sum_{i\,j}=\rho \,u_{i}\,u_{j}+\left(p-p_{0}\right)\,\delta_{i\,j}-\tau_{i\,j}.$$

The Actran software computes two source terms, volume and surface contributions, using unsteady fluid velocity and density fields saved in the CFD files that relate to the acoustic mesh (Free Field Technologies, 2020). Because the vascular wall was set to be rigid, the normal acceleration was zero, so the boundary integral vanished. Thus, only the volume source term was considered in this study.

### Doppler Auscultation and Sonification of Flow Sound

Doppler auscultation of the flow sound and CFD acoustics were measured and sampled at the transverse–sigmoid mainstream sinus flow/node 11,131 and the center of the diverticula/node 13,995 of case 3. An acoustic file containing three cycles of duplicated representative CFD outcomes was manually created using Adobe Audition cc 2020 (Adobe Inc.) for likeness rating purposes. The participants were asked to rate the likeness of the acoustic files using a visual analog score from 0 to 10. Sonification of the PT was performed in accordance with our previous reports using MATLAB R2017a (MathWorks). To visualize the frequency component of the recorded venous sound, a short-time Fourier transform [Eq. (9)] was implemented:


(9) $$\text{STFT}\,\left\{x\,\left[n\right]\right\}\,\left(m,\omega \right)\,X\,\left(\tau ,\omega \right)=\sum_{n=-\infty }^{\infty }x\,\left[n\right]\,w\,\left[n-m\right]\,e^{-j\,\omega \,n},$$

where *x*[*n*] represents the sequence of discretized time-domain signals to be transformed, *m* is the time index, ω is the frequency, and *w*[*n*] denotes the sequence of discretized window functions. The amplitude of the acoustic data was normalized for data comparison.

### Statistical Analysis

Statistical analysis was performed using RStudio software (RStudio, Boston, MA, United States) and Origin 9.1.0 Pro (OriginLab Corporation, Northampton, United States). The normality of continuous data was checked using the Shapiro–Wilk test. A two-sample *t*-test or the Mann–Whitney *U* test was performed based on the outcome of data normality, as appropriate. The Pearson or Spearman correlation coefficients were computed to examine the correlations among the objective measurements. Based on Cohen’s criteria (Cohen, 1988), the correlation strength was defined as very strong (0.5–1.0), moderate (0.3–0.5), or weak (<0.3). Statistical significance was set at *P* < 0.05.

## Results

### Clinical and Radiological Characteristics

Nineteen participants (3 men and 16 women) were enrolled in this study. Most of these participants had right-sided PT (*n* = 14, 73.6%). The mean age of the participants was 36.9 ± 8.2 years. The mean PT duration was 53.0 ± 58.5 months, and the shortest and longest duration of PT were 2 and 204 months, respectively.

Of the 19 cases, 18 of them (94.7%) had PT on the dominant side of the transverse–sigmoid sinus system. The remaining patient had a co-dominant bilateral transverse–sigmoid sinus system. The average normalized degrees of TSS on the PT and contralateral sides were 71.6 ± 14.2% (one case < 33%, three cases = 33–66%, and 13 cases > 66%) and 72.4 ± 11.0% (three cases = 33–66% and five cases > 66%), respectively. Contralateral hypoplastic transverse sinuses were found in 11 (57.8%) cases. The average ITSS was 9.4 ± 2.9. An empty sella was found in eight (42.1%) participants.

### Results of Retroauricular Color-Coded Doppler

**FIGURE 5:**
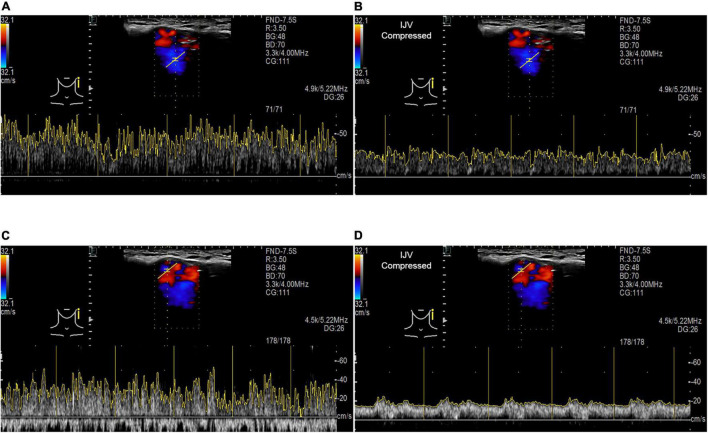
RCCD and IJV compression examination of the association between PT and transverse–sigmoid junction hemodynamics. IJV indicates the internal jugular vein. The yellow vertical lines indicate the RR intervals of the electrocardiogram. **(A)** Real-time mainstream sinus flow velocity spectrum before IJV compression. **(B)** Real-time mainstream sinus flow velocity spectrum during IJV compression. **(C)** Real-time diverticular flow velocity spectrum before IJV compression. **(D)** Real-time diverticular flow velocity spectrum during IJV compression.

When the ipsilateral IJV was compressed, the PT completely disappeared in all participants, and the vortex inside the diverticulum persisted in 18 out of 19 patients. The remaining patients’ mainstream sinus flow and diverticular flow velocity were undetectable, that is, reduced to zero. The figures and data of RCCD hemodynamics are shown in Figure 5 and Table 2. The median *V*_mn_ of the mainstream sinus flow and the diverticular flow was 40.2 (28.7/48.1) cm/s and 46.0 (34.4/63.0) cm/s, respectively, whereas after ipsilateral IJV compression, the median *V*_mn_ of the mainstream sinus flow and the diverticular flow dropped to 20.6 (13.4/25.5) cm/s (38.1%) and 23.3 (20.1/36.8) cm/s (39.8%). The median *V*_max_ of the mainstream sinus flow and the diverticular flow was 58.8 (45.2/64.5) cm/s and 70.6 (53.7/106.8) cm/s, respectively, whereas the median *V*_max_ of the mainstream sinus flow and the diverticular flow was 37.6 (25.0/43.5) cm/s and 49.8 (29.4/58.9) cm/s, respectively, when the ipsilateral IJV was compressed. There were significant differences in the reduction of *V*_mn_ (two-sample *t*-test, *p* < 0.01) and *V*_max_ (Mann–Whitney *U* test, *p* < 0.01) of the mainstream sinus flow. Additionally, a significant reduction in the *V*_mn_ (Mann–Whitney *U* test, *p* < 0.01) and *V*_max_ (Mann–Whitney *U* test, *p* = 0.01) of the diverticular flow was found. The median resistive index of the mainstream sinus flow and the diverticular flow was 0.60 (0.57/0.75) and 0.50 (0.41/0.69), respectively. The mean pulsatility index of the mainstream sinus flow and the diverticular flow was 1.01 (0.66/1.32) and 0.72 (0.60/1.27), respectively. No statistical significance was found in the resistive and pulsatility indices when the ipsilateral IJV was compressed.

**TABLE 2:** Results of RCCD transverse–sigmoid junction hemodynamics of 19 participants.

Mainstream sinus flow				

	Mean velocity	Peak velocity	Resistive index	Pulsatility index
	cm/s	cm/s	Dimensionless	Dimensionless
RCCD	40.2 (28.7/48.1)	58.8 (45.2/64.5)	0.60 (0.57/0.75)	1.01 (0.66/1.32)
RCCD while IJV compression	20.6 (13.4/25.5)	37.6 (25.0/43.5)	0.60 (0.51/0.78)	0.98 (0.58/1.30)
*p* value	<0.01^a^	<0.01^b^	0.953^b^	0.860^a^
Diverticular flow				
	Mean velocity	Peak velocity	Resistive index	Pulsatility index
	cm/s	cm/s	Dimensionless	Dimensionless
RCCD	46.0 (34.4/63.0)	70.6 (53.7/106.8)	0.50 (0.41/0.69)	0.72 (0.60/1.27)
RCCD while IJV compression	23.3 (20.1/36.8)	49.8 (29.4/58.9)	0.55 (0.39/0.65)	0.73 (0.54/1.16)
*p* value	<0.01^b^	0.01^b^	0.831^a^	0.881^b^

*Variables are expressed as median and interquartile range. RCCD, retroauricular color-coded Doppler method.*

*^a^Two-sample t-test; ^b^Mann–Whitney U-test.*

The results of the correlation between hemodynamics, normalized TSS degree, and ITSS are shown in Table 3. There was no significant correlation among *V*_mn_, *V*_max_, and the degree of ipsilateral TSS, indicating high heterogeneity in the ipsilateral mainstream sinus and diverticular hemodynamics among subjects with venous PT.

**TABLE 3:** Results of correlation analysis between hemodynamics and transverse sinus stenosis.

Ipsilateral normalized TSS	Mainstream sinus flow		Diverticular flow	
	Mean velocity	Peak velocity	Mean velocity	Peak velocity
Spearman *r* value	0.145	0.203	– 0.115	– 0.02
Spearman *p* value	0.551	0.403	0.636	0.914
**ITSS score**				
Spearman *r* value	0.225	0.249	0.167	0.290
Spearman *p* value	0.353	0.302	0.494	0.226

*TSS, transverse sinus stenosis; ITSS, index of transverse sinus stenosis.*

*All correlation statistics were done using the Spearman correlation.*

### Bilateral Internal Jugular Vein Outflow

The complete hemodynamic data of the bilateral IJV are shown in Table 4. The ipsilateral IJV outflow volume was 10.0 (6.9/11.8) g/s, and the contralateral IJV outflow volume was 3.6 (2.4/7.7) g/s. There was a statistically significant difference between the ipsilateral and contralateral IJV outflow volumes (Mann–Whitney *U* test, *p* = 0.012). The median *V*_mn_ of the ipsilateral and contralateral IJV flow was 20.5 (15.5/24.7) cm/s and 22.7 (13.9/32.3) cm/s, respectively, (Mann–Whitney *U* test, *p* = 0.89). The median *V*_max_ of the ipsilateral and contralateral IJV was 27.9 (23.9/41.6) cm/s and 30.2 (21.4/46.3) cm/s, respectively, (Mann–Whitney *U* test, *p* = 0.782). The resistive index of ipsilateral and contralateral IJV flow was 0.45 (0.27/0.69) and 0.46 (0.33/0.64), respectively, (Mann–Whitney *U* test, *p* = 0.629). The pulsatility index of the ipsilateral and contralateral IJV flow was 0.64 (0.33/1.12) and 0.78 (0.41/1.12), respectively, (Mann–Whitney *U* test, *p* = 0.565).

**TABLE 4:** Results of bilateral internal jugular vein hemodynamics of 19 participants.

	Outflow volume	Mean velocity	Peak velocity	Resistive index	Pulsatility index
	g/s	cm/s	cm/s	Non-dimensional	Non-dimensional
Ipsilateral IJV	10.5 (9.3/17.9)	22.1 (16.9/31.9)	31.5 (25.2/44.2)	0.44 (0.27/0.62)	0.63 (0.33/0.99)
Contralateral IJV	4.7 (2.6/7.4)	24.4 (14.9/34.5)	33.8 (22.6/47.3)	0.46 (0.29/0.68)	0.70 (0.35/1.14)
*p* value	<0.01	0.792	0.609	0.682	0.619

*Variables are expressed as median and interquartile range.*

*IJV, internal jugular vein.*

*All statistics were done using the Mann–Whitney U-test.*

### Hydroacoustic Outcome

**FIGURE 6:**
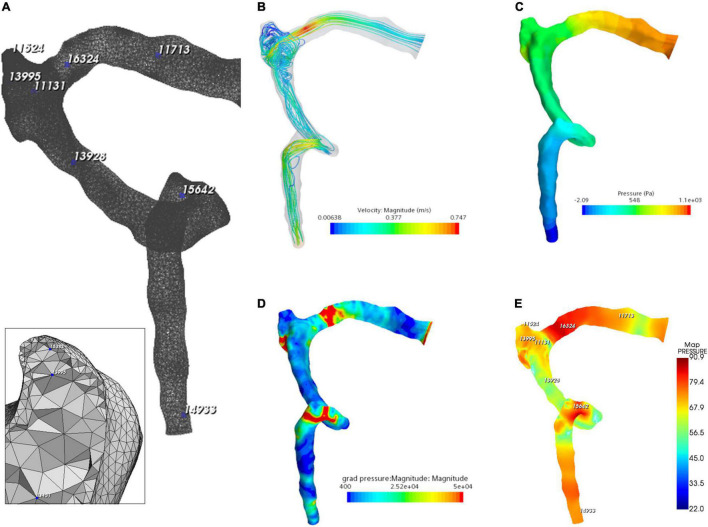
Hydroacoustic characteristics of the ipsilateral transverse–sigmoid sinus. **(A)** A 3D finite element model of ipsilateral transverse–sigmoid sinus of case 3. Eight nodes were chosen for hydroacoustic evaluation. **(B)** Velocity streamline of the ipsilateral transverse–sigmoid sinus. **(C)** Wall pressure distribution of the ipsilateral transverse–sigmoid sinus. **(D)** Pressure gradient distribution of the ipsilateral transverse–sigmoid sinus (unit: Pa/m). **(E)** Acoustic distribution of the ipsilateral transverse–sigmoid sinus (unit: dBA).

The flow visualization and hemodynamics of the CFD results are shown in Figure 6. The peak velocity of the flow field was 0.74 m/s at the TSS. The TSS, the anteroinferior portion of the diverticulum, and the jugular bulb were regions with larger pressure gradients. The peak pressure gradient of the ipsilateral transverse–sigmoid sinus was 441,407.7 Pa/m. In contrast to the pressure gradient, the wall pressure of the transverse–sigmoid sinus gradually decreased from the inlet to the outlet, where the peak wall pressure was 1,097.9 Pa. Regarding the diverticulum, the greatest wall pressure appeared at the anteroinferior surface, which coincided with the location of dehiscence.

**FIGURE 7:**
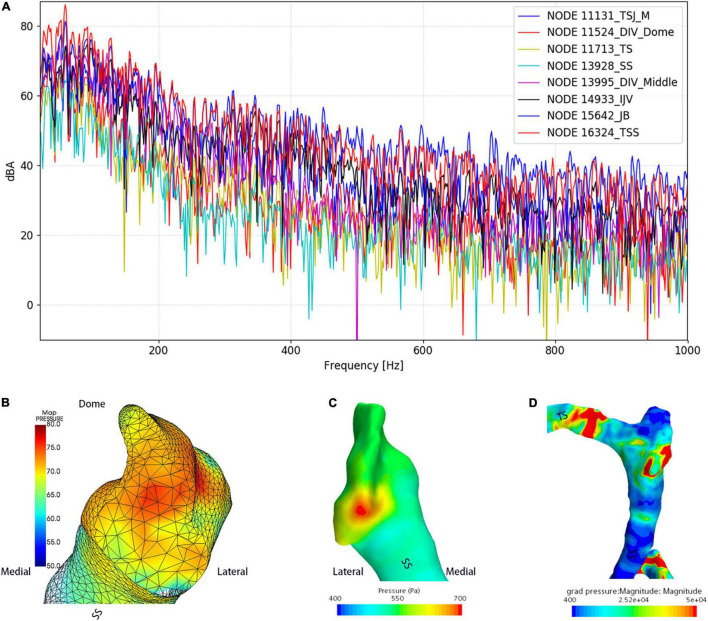
Regional hydroacoustic characteristics of the ipsilateral transverse–sigmoid sinus. SS indicates the sigmoid sinus. **(A)** The amplitude–frequency spectrum of the ipsilateral transverse–sigmoid sinus. **(B)** The amplitude of the diverticular flow (unit: dBA). **(C)** Wall pressure distribution at the transverse–sigmoid junction and the diverticulum. **(D)** Pressure gradient distribution of the ipsilateral transverse–sigmoid sinus.

The results of CFD acoustics are shown in Figure 7 and Table 5. The largest peak amplitude was 86.2 dBA, found in the TSS region. The TSS also presented the largest RMS amplitude of 67.7 dBA. In contrast, the peak and RMS amplitude of the upper curve sigmoid sinus were 65.7 and 48.0 dBA, respectively, which were the lowest among the observed anatomical locations. The largest difference in the flow amplitude was 20.5 dB. The peak amplitude at the middle diverticulum was 0.9 dBA larger than the mainstream transverse–sigmoid sinus flow, whereas the RMS amplitude of the mainstream transverse–sigmoid sinus flow was 1.8 dBA. The frequency of the largest peak amplitude ranged from 49.4 to 92.5 Hz.

**TABLE 5:** Results of CFD hydroacoustic outcomes.

Locations	TS	TSS	TSJ. M.	Div. Mid.	Div. dome	SS	JB	IJV
Node number	11,713	16,324	11,131	13,995	11,524	13,928	15,642	14,933
Peak amplitude (dBA)	72.5	86.2	74.3	75.2	71.4	65.7	81.4	74.9
Frequency at peak amplitude (Hz)	58.6	58.6	60.1	49.4	58.6	63.2	58.6	92.5
RMS amplitude* (dBA)	52.3	67.7	57.1	59.9	53.2	48.0	63.7	59.3

*TS, transverse sinus; TSS, transverse sinus stenosis; TSJ. M., transverse–sigmoid junction mainstream; Div.Mid., middle portion of the diverticulum; Div. Dome, the dome of the diverticulum; SS, sigmoid sinus; JB, jugular bulb; and IJV, internal jugular vein.*

*RMS amplitude measured from 0 to 1 kHz.

**FIGURE 8:**
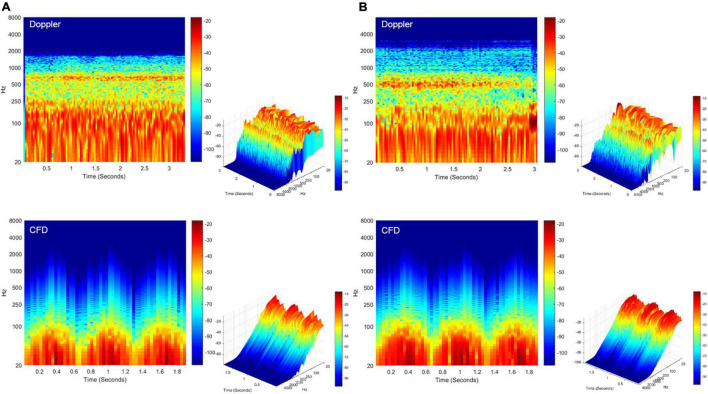
Spectrotemporal results of *in vivo* Doppler and CFD. **(A)** Short-time Fourier transformation of the Doppler mainstream sinus flow (upper panel) and CFD mainstream sinus flow (lower panel, node 11131). **(B)** Short-time Fourier transformation of the Doppler diverticular flow (upper panel) and CFD diverticular flow (lower panel, node 13995). CFD, computational fluid dynamics.

The short-time Fourier transforms of the Doppler and CFD acoustic data are shown in Figure 8. The patient-based likeness scoring of the *in vivo* Doppler transverse–sigmoid mainstream sinus (Supplementary Material 1) and diverticular flows (Supplementary Material 2) were 7 and 5, whereas the ratings of CFD acoustics of the corresponding locations were 6 (Supplementary Material 3) and 6 (Supplementary Material 4), respectively.

## Discussion

The RCCD technique is a newly proposed Doppler ultrasound method for detecting mainstream sinus and diverticular hydroacoustic characteristics at the transverse–sigmoid junction. By conflating the RCCD technique with ipsilateral IJV compression, the changes between PT and *in vivo* hemodynamics can be investigated non-invasively. It was discovered that PT subsided when the *V*_mn_ of sinus flow reduced by approximately 50%. From the presence to the disappearance of PT, it is postulatsed that this quantitative range of flow velocity reduction offers a bottom-line curative marker for both extraluminal compression and endoluminal surgeries. However, the former surgical technique conceivably requires less flow velocity reduction due to the thickening of the soundproof sigmoid wall, although the compression depth of the transverse–sigmoid junction ought to be performed with circumspection to prevent the iatrogenic cause of intracranial pressure (Hsieh et al., 2022; Qiu et al., 2022). A recent clinical study revealed that sinus flow velocity is reduced after a lumbar puncture without a concomitant reduction in the bulk flow rate, resulting in a 3.8 ± 3.4 improvement of PT intensity on a 0–10 Likert scale in 10 subjects (Haraldsson et al., 2019). They hypothesized that velocity, not flow rate, was related to PT. However, in this study, planar velocity and flow volume were reduced at the transverse–sigmoid junction when the PT was completely suppressed by ipsilateral IJV compression. Furthermore, the ipsilateral sinus outflow volume was significantly larger than that of the contralateral side. Although the contralateral *V*_mn_ of the IJV was found to be slightly higher than that of the ipsilateral side, given the constant negative pressure produced by the heart during the diastolic phase, the flow velocity of the returning venous flow was conceivably determined by the cross-sectional area tangential to the flow direction. Regarding previous clinical discoveries, it is not uncommon for PT to intensify when the contralateral IJV is compressed, and in subjects with bilateral SSWAs, unilateral PT may alter to the contralateral side during compression of the ipsilateral IJV (Kline et al., 2020; Hsieh et al., 2021b). This signifies that the asymmetric distribution of the flow volume may underpin the development of PT. Thus, we extrapolate that a high flow velocity and flow volume, that is, a high flow kinetic energy, are prerequisite conditions for PT.

Due to the fluctuating shape of the transverse–sigmoid sinus, differences in the sinus flow velocity and pressure gradient distribution led to a segmental distribution of flow amplitude. A larger amplitude of the flow sound was detected at segments with a higher flow velocity and pressure gradient. This finding substantiates the therapeutic effect of endoluminal TSS stenting, which targets the reduction of regional flow velocity and relief of the *trans*-stenotic pressure gradient (Mu et al., 2021a). Previous computational studies found that the flow-induced displacement of the vascular wall could generate vibro-acoustic noise from 48.76 to 116.57 dB (Tian et al., 2017; Mu et al., 2021a,b). The amplitude of the vibro-acoustic sound surpassed that of the hydroacoustic source when the volume and surface contributions were juxtaposed (Hsieh et al., 2022). Nevertheless, based on intraoperative measurements, the frequency of vascular displacement was below the hearing threshold (Hsieh et al., 2022). It remains unclear whether the vibro-acoustic sound is generated solely from the vibration of the sigmoid sinus wall. To that end, PT likely results from hydroacoustic noise in the junction with the vibration of the vascular wall.

The vortex has been suggested to cause PT in subjects with SSWAs (Amans et al., 2018; Eisenman et al., 2018). A large 4D flow MR case series found that 68% of subjects with diverticulum presented a vortex (Li et al., 2021). In contrast to the mainstream sinus flow, RCCD unveiled that the vortex inside the diverticulum is characterized by low or no pulse synchronicity, and the hydroacoustic sound generated by the vortex is less similar to the participants’ PT. The PT disappears despite the vortex remaining inside the diverticulum. This strongly indicates that the flow kinetic energy outweighs the formation of vortices in the acoustic production of the PT. In addition, a difference of less than 1 dB was detected by virtual microphones placed at the center of the mainstream and diverticular flows simulated using the CFD method. These results imply that the flow velocity outweighs the vortex in the acoustic production of PT. In fact, individuals with a diverticulum can be accidentally identified even in the absence of PT (Hsieh et al., 2021b). Furthermore, the therapeutic effect of complete reduction of a diverticulum can be unreliable intraoperatively, and PT can be resolved by reducing the flow velocity even without the exclusion of the diverticulum from venous return (Han et al., 2017; Zhang et al., 2019; Hsieh and Wang, 2020). Based on the current RCCD results and previous incidences, a diverticulum is not an essential prerequisite for PT.

Transcranial Doppler ultrasound reveals intracranial real-time flow hydroacoustics using the Doppler wave effect, which yields higher spatial and temporal resolutions than MR techniques (Meckel et al., 2013). Studies have suggested that MR methods tend to underestimate the peak systolic velocity of intracranial and cervical arteries compared to ultrasound (Harloff et al., 2013). Compared to the only previous imaging study that investigated subjects with diverticulum independently, the *V*_mn_ detected by the current RCCD technique closely conforms to the results measured by 4D MR (Li et al., 2021), with 7.3% difference in *V*_mn_ (Table 6); nonetheless, a stark difference in *V*_max_ is found between their and our results. Additionally, ITSS in this study was also found uncorrelated with the hemodynamic parameters, which is antithetical to the study of Ding et al. (2021), since the Doppler insonation plane was more distant from the center of TSS, from which the sinus flow velocity can decelerate after issuing from a TSS. We reckon that these study discrepancies predominantly result from the fundamental difference in the selection of region/point of interest. Hence, the planar hemodynamic parameters gaged by the RCCD method can yield crucial hemodynamic insights and allow surgeons to cope with PT objectively.

**TABLE 6:** Comparison of results in subjects with diverticulum.

	Number of subjects with diverticulum	*V*_mn_ (cm/s)	*V*_max_ (cm/s)	Measurement plane
Previous 4D-MR Study [1]	22	55.92 ± 20.08 [1]	147.58 ± 42.16 [1]	*V*_max_ at TS-SS [1]
Present study	19	52.08 ± 28.61^a^	85.14 ± 48.55^a^	TSJ

*^a^Largest value acquired from the TSJ region expressed in mean ± standard deviation.*

*TS-SS, transverse–sigmoid sinus; TSJ, transverse–sigmoid junction; and V_max_, peak velocity.*

*Comparison of our results with previously reported results: [1] Data from Li et al. (2021).*

The benefits of emerging 4D MR techniques for detecting the hemodynamics of dural venous sinus flows, however, are promising and conspicuous in comparison to ultrasound techniques. As the 4D MR technique allows 3D coverage of the transverse–sigmoid sinus, the possibility of measuring flow velocities at any time/spatial point of interest after scanning can be achieved. Amans and Wang/Gong’s research teams have combined 4D flow MR techniques with CFD methods (Amans et al., 2018; Haraldsson et al., 2019; Ding et al., 2021; Li et al., 2021). It has been suggested that highly convoluted flow patterns are observed in those with a diverticulum (Amans et al., 2018; Li et al., 2021). Furthermore, post-stenotic high jet flow velocity and *trans*-stenotic pressure gradient near the transverse–sigmoid junction, both of which are subjected to variations in intracranial pressure (Haraldsson et al., 2019), may be considered as the salient contributing factors of PT (Ding et al., 2021). The *in vivo* RCCD indicates that immediate reduction of flow velocity at the transverse–sigmoid junction is directly correlated to the elimination of PT. However, it remains unknown whether PT arises owing to the formation of dehiscence or the fact that intracranial hemodynamics are altered in the first place.

This study is limited by the sample size for the RCCD examination. However, the current sample size is on par with the previous 4D-MR flow or CT venogram studies. As this study focuses on intracohort comparisons rather than group comparisons, the current Doppler results appear to demonstrate a convincing reduction in flow velocity modulated by ipsilateral IJV compression. Furthermore, RCCD is reserved for those with a protrusive diverticulum or laterally placed sigmoid sinus, which hampers the understanding of PT in patients with a complete mastoid cortex and in healthy controls. The TCCD technique can provide a high temporal and spatial resolution of the transverse sinus flow spectrum for CFD simulation, which benefits hydroacoustic investigations of the entire transverse–sigmoid sinus system. However, the poor temporal window can greatly reduce insonation of the contralateral skull base and sampling of the blood flow. Thus, we are currently working on increasing the TCCD data to reveal differences between subjects with and without PT. Although the current computational acoustic data reaches a high resemblance of PT sound, nuanced acoustic differences between Doppler and current CFD techniques require a thorough and comprehensive investigation in the future. Continuous refinement of the establishment of boundary conditions is warranted.

## Conclusion

The currently proposed RCCD method unfolded transient hydroacoustic characteristics of sinus flow at the transverse–sigmoid junction with high spatial and temporal resolutions. By implementing the RCCD method in junction with ipsilateral IJV compression, PT subsides as the *V*_mn_ of the mainstream sinus flow and the diverticular vortex decreases averagely by 51.2 and 50.6%, respectively. However, the diverticular vortex persisted during ipsilateral IJV compression, notwithstanding a holistic reduction in sinus flow kinetic energy. In addition, the amplitude difference between the mainstream sinus and diverticular flows was less than 1 dB based on our CFD analysis. Therefore, PT is associated with the flow kinetic energy instead of the sheer formation of vortex *per se*; regions with larger flow velocity and pressure gradient engender greater flow amplitude. As the combined implementation of the RCCD technique and ipsilateral IJV compression uncloaks individual quantitative range of velocity reduction to eliminate PT non-invasively, this marker can be applied as a presurgical curative index for surgeons to resolve PT efficiently.

## Data Availability Statement

The original contributions presented in this study are included in the article/Supplementary Material, further inquiries can be directed to the corresponding author.

## Ethics Statement

The studies involving human participants were reviewed and approved by Ethical Committees of the Eye, Ear, Nose, and Throat Hospital in Shanghai, China. The patients/participants provided their written informed consent to participate in this study.

## Author Contributions

XG performed all Doppler examinations and drafted the article. Y-LH designed the study, assisted in drafting the manuscript, and performed all statistical, radiologic, and acoustic analyses in this study. XW performed computational fluid dynamics and post-analysis. WW is the lead surgeon who supervised this study. All authors contributed to the article and approved the submitted version.

## Conflict of Interest

The authors declare that the research was conducted in the absence of any commercial or financial relationships that could be construed as a potential conflict of interest.

## Publisher’s Note

All claims expressed in this article are solely those of the authors and do not necessarily represent those of their affiliated organizations, or those of the publisher, the editors and the reviewers. Any product that may be evaluated in this article, or claim that may be made by its manufacturer, is not guaranteed or endorsed by the publisher.
